# IHIBE: A Hierarchical and Delegated Access Control Mechanism for IoT Environments

**DOI:** 10.3390/s24030979

**Published:** 2024-02-02

**Authors:** Hari Purnama, Masahiro Mambo

**Affiliations:** 1 Division of Electrical Engineering and Computer Science, Graduate School of Natural Science and Technology, Kanazawa University, Kanazawa 920-1192, Japan; 2 Institute of Science and Engineering, Kanazawa University, Kanazawa 920-1192, Japan

**Keywords:** Internet of Things, access control, IOTA, hierarchical identity-based encryption, HIBE, hierarchical access control

## Abstract

Ensuring authorized access control in the IoT is vital for privacy and safety protection. Our study presents the novel IHIBE framework, which combines IOTA (a distributed ledger technology) with hierarchical identity-based encryption (HIBE), thereby enhancing both IoT security and scalability. This approach secures access tokens and policies while reducing the computational demand on data owners. Our empirical findings reveal a significant performance gap, with access rights delegation on the Raspberry Pi 4 exceeding those on AWS by over 250%. Moreover, our analysis uncovers optimal identity policy depths: up to 640 identities on AWS and 640 on the Raspberry Pi 4 for systems with higher tolerable delays, and 320 identities on AWS versus 160 on the Raspberry Pi 4 for systems with lower tolerable delays. The system shows practical viability, exhibiting insignificant operational time differences compared to Zhang et al.’s schemes, particularly in access rights verification processes, with a minimal difference of 33.35%. Our extensive security assessment, encompassing scenarios like encrypted token theft and compromise of authority, affirms the efficacy of our challenge-response and last-word challenge (LWC) mechanisms. This study underscores the importance of platform choice in IoT system architectures and provides insights for deploying efficient, secure, and scalable IoT environments.

## 1. Introduction

The term “Internet of Things” (IoT) refers to the idea of various devices and objects being interconnected over the Internet. The IoT comprises linked sensors, processors, and actuators, all working to deliver a specific service. The IoT facilitates human-to-human, human-to-machine, and machine-to-machine interactions by implementing identification, management, and control procedures [1]. The rapid increase in IoT adoption can be attributed to its use in various sectors, such as the military, government, agriculture, smart cities, industry, education, and healthcare.

However, IoT devices, particularly sensors, are frequently designed with little regard for security, leading to serious security problems. Unauthorized access to IoT resources is a significant security problem that has been widely highlighted [2]. Given the proximity of IoT devices to individuals and the personal nature of the data they handle, securing access to IoT resources is critical to protect our privacy, property, and safety.

Access control refers to the limitation of resource accessibility solely to authorized individuals. Modern access control systems typically centralize the storage of policies that define ‘which users can access which resources’ for administrative ease. However, this centralization poses a risk, as the server becomes a single point of failure vulnerable to destruction by disasters or breaches by malicious actors. Furthermore, centralized servers may experience load concentration in massive systems like the IoT. As a result, distributed backups for access policies, stability, and scalability requirements are needed for access control in IoT systems.

IoT device data collection generates vast amounts of information that is extremely valuable for big data mining, analytics, and analysis. However, this promise depends on the availability of a sizable amount of data owned by IoT users and stored both in the cloud and at the edge (such as sensors) [3]. The reliability of trusted third parties, which securely hold data and perform access control by keeping an access control list (ACL), is a common foundation for current data sharing and user privacy models.

This study identifies the implicit resource and user hierarchies within each access relation. Since some users have more access rights than others, and some resources require more access restrictions, hierarchies logically result from these differences in access rights and limitations (a detailed explanation will be given later). This study demonstrates how these hierarchies can provide insightful data [4].

IoT systems that use hierarchical-based access control must be scalable, reliable, and have distributed backups of access policies. Frameworks for distributed hierarchical-based access control utilizing IOTA technology have been proposed to satisfy these needs. IOTA can be compared to a distributed database administered using peer-to-peer (P2P) networks. IOTA is an open-source, scalable distributed ledger platform that offers real-time microtransactions and transactions using the Tangle data structure. With no intermediary or central administrator, the node network of IOTA duplicates, shares, and synchronizes digital data and value dispersed across numerous places. The node facilitates the secure interchange of currency and data without costs. Each peer records a copy of the transaction and confirms its validity. IOTA is ideal for storing access rights and rules because of its dispersed and tamper-resistant features.

The utilization of IOTA was proposed as a means to regulate access control in [5]. A cryptographic token is securely stored within the Tangle, utilizing distributed and tamper-resistant methods. Nevertheless, there is an underlying assumption that the exchange of information among entities is safeguarded. The mechanism still needs to be improved by offering one-to-many access control. The secure approach presented in [6] employs ciphertext policy attribute-based encryption (CP-ABE). The token is encrypted using CP-ABE and subsequently stored in the Tangle, providing streamlined token management through one-to-many encryption.

The main contribution of this paper is the presentation of IHIBE (IOTA with hierarchical identity-based encryption), a refined framework for access control in IoT environments within hierarchical organizations, addressing the limitations of the CP-ABE scheme. Unlike CP-ABE, which does not fully account for real-world IoT scenarios, our model emphasizes the importance of adhering to hierarchical organization. Our framework introduces a novel aspect of securing not only the access tokens but also the policies themselves. Our framework cascades these policies from the central authority to domain authorities, securing them to prevent lower-level entities from accessing upper-level policies. In IHIBE, each IoT device within a domain must comply with the authority-defined policies, ensuring a structured approach to identity management. IoT environments represent identities by key attributes, such as location, without excessive detail, aligning with the practicality of this setup. The key innovation is reducing the data owner’s burden by shifting setup and key generation responsibilities to the central authority or domain authorities at various hierarchy levels. The data owner is responsible for encrypting the data and conducting the verification process for data requesters who want to access the device or the data. By focusing on a fixed set of identities within the system and securing tokens, policies, and data, IHIBE offers a robust solution to access control, reducing the burden on data owners and ensuring secure and efficient operation in hierarchical IoT environments.

Integrating hierarchical identity-based encryption (HIBE) with IOTA significantly benefits data management, primarily because of its robust data integrity; storing data on the Tangle makes it exceedingly difficult to alter, ensuring reliable and trustworthy information. IOTA’s high availability and scalability make it well suited for efficiently managing vast volumes of IoT data. Security is another cornerstone of IOTA’s architecture, offering strong protections against various threats, a critical aspect of IoT systems.

To illustrate the hierarchies, we will use a simple example inspired by university-level management. In summary, this paper’s main contribution is the introduction of a hierarchical access control mechanism with a key delegation process. As far as we know, this is the first research to implement HIBE’s key delegation process in IOTA.

The structure of this paper is organized in the following manner. Section 2 offers insights into the existing research relevant to our topic. Explanations of the fundamental concepts, including IOTA, the CP-ABE scheme, the limitations of CP-ABE, and the IHIBE scheme, are given in Section 3. The details of our proposed system are elucidated in Section 4, followed by an exposition of its implementation in Section 5. Our system’s performance is evaluated in Section 6. Finally, Section 7 summarizes the key points of our study.

## 2. Related Works

### 2.1. Conventional Access Control

Access control is crucial in the security field. There are several categories of access control, namely preventive, detective, corrective, recovery, deterrent, and compensating. It involves two aspects: physical and logical. Security precautions are provided by access control to limit a subject’s access to an object. The creation of an access control mechanism, therefore, encompasses identity, authorization, and authentication. The subject or user can use credentials to obtain authentication during the identification step. After submitting valid credentials, the user is permitted access only to those resources given by an administrator (or resource owner) through access control permissions.

The fundamental concept of this model is to define a set of rules that determine who is permitted to access which system resources. Typically, these rules are based on the user’s identity, group, and the permissions associated with the resource. In the conventional access control paradigm, users are generally assigned to one or more groups associated with specific access permissions. Individual files, directories, and other system resources can be granted read, write, execute, and delete permissions [7].

Various access control frameworks, including access control lists (ACLs), discretionary access control (DAC), role-based access control (RBAC), identity-based access control (IBAC), and attribute-based access control (ABAC), play a crucial role in safeguarding the availability, integrity, and confidentiality of resources. ACLs establish user or group access permissions for system resources, setting the groundwork for access-level management [8]. In contrast, RBAC systems allocate access permissions based on roles like administrator or manager, aligning access rights with the user’s professional responsibilities [9]. Identity-based access control is a method of controlling access, where the rights and restrictions of users in a system are determined based on their identities and/or associated attributes [10]. ABAC approaches determine access permissions through attributes like user identity, geographical location, time of access, and the type of device used [11]. Furthermore, capability-based access control (CapBAC) reinforces this spectrum by issuing tokens to validate specific access rights [12], thus adding another layer of complexity to access control in computing environments.

Although they are derived from classic models, traditional access control systems frequently necessitate modernization to adeptly handle large-scale access control. These systems are commonly designed to centralize the repository and administration of access rights and policies on a single server, inadvertently establishing a single point of failure. This centralization renders the system susceptible to disruptions caused by natural disasters or deliberate user attacks [13,14]. Such attacks compromise access rights, potentially leading to unauthorized resource access. Furthermore, in expansive network settings, the central server may struggle to process a growing volume of access requests, which can impede system performance. Therefore, there is an urgent requirement for access control techniques that are distributed, robust, and scalable to keep pace with the burgeoning growth of IoT networks.

### 2.2. Blockchain-Based Access Control Scheme

An Ethereum-based distributed ABAC system, in which the properties of the subjects, objects, and access control rules are recorded to smart contracts by the appropriate administrators, was proposed in [15]. The Ethereum-based RBAC scheme uses smart contracts to maintain associations between subjects and roles and between roles and access permits [16]. Using the relationships that have been stored, resource owners can choose which subjects can access the resource in this manner. When an object owner receives an access request from a subject, the related smart contract is triggered to check the subject’s access rights. The intelligent system uses attributes and policies to control access and provides the object owner with the verification outcome. Utilizing intelligent contracts enables the reliable management of decision making and access rights. Additionally, due to the decentralized nature of the functions, access control can be enforced even when some peers behave strangely. Access control models based on other models, like ACL, or different blockchain platforms, like Bitcoin, can be found in [17,18].

Similarly, the work presented in [19] explored blockchain integration with identity-based encryption for managing digital identities, proposing a blockchain solution where public digital identities are linked to transactions through IBE. This approach enhances the blockchain’s ability to manage access control by ensuring that the identity of the transaction signers is directly associated with their cryptographic keys, aligning with the eIDAS regulation for digital identification. Adding to this perspective, ref. [20] introduced a hierarchical approach to blockchain-based access control in the IoT. By leveraging a decentralized hierarchical identity-based signature scheme, HIBEChain addresses key management and accountability issues, showcasing a scalable and secure solution for managing access in large IoT networks. This novel system complements existing blockchain access control models by offering a structured and efficient way to handle identity verification and access management in expansive IoT settings. Further enriching this discourse, ref. [21] examined the application of identity-based encryption in enhancing secure communication across different blockchain platforms in the IoT. This study highlights the role of IBE in facilitating secure data transmission and cross-chain interactions, which are integral to effective access control in blockchain-based IoT environments.

### 2.3. IOTA-Based Access Control Scheme

In distributed ledger technologies, IOTA stands out for enabling feeless microtransactions. A decentralized capability-based access control framework using IOTA (DCACI) was presented in [5]. Subject access rights are encapsulated as tokens within IOTA’s Tangle, a distributed ledger similar to blockchain, ensuring a secure and distributed storage mechanism. Despite its benefits, DCACI encounters challenges related to scaling and security, particularly in managing tokens, and needs in-depth security features. Addressing these issues, the research presented in [6] applied IOTA to enhance the DCACI framework, aiming for greater flexibility, finer granularity, and improved scalability in access control. The implementation uses CP-ABE, allowing for a more flexible and secure authorization process. While akin to DCACI in its use of tokens for subject authorization, this system differs in that these tokens are distributed through the Tangle after encryption with CP-ABE. This method, defined by CP-ABE policies, achieves more intricate and detailed access control, boosts scalability, and eases the burden of token management for object owners.

In this context, ref. [22] provided a critical perspective by discussing a system that merges data integrity and access management in a decentralized setup, integrating IOTA’s Tangle with the InterPlanetary File System (IPFS). This approach enables secure data sharing and necessitates a robust access control mechanism, thus aligning with the IoT’s advanced access control frameworks, as showcased in IOTA-based schemes.

## 3. Preliminaries

### 3.1. IOTA

**Figure 1:**
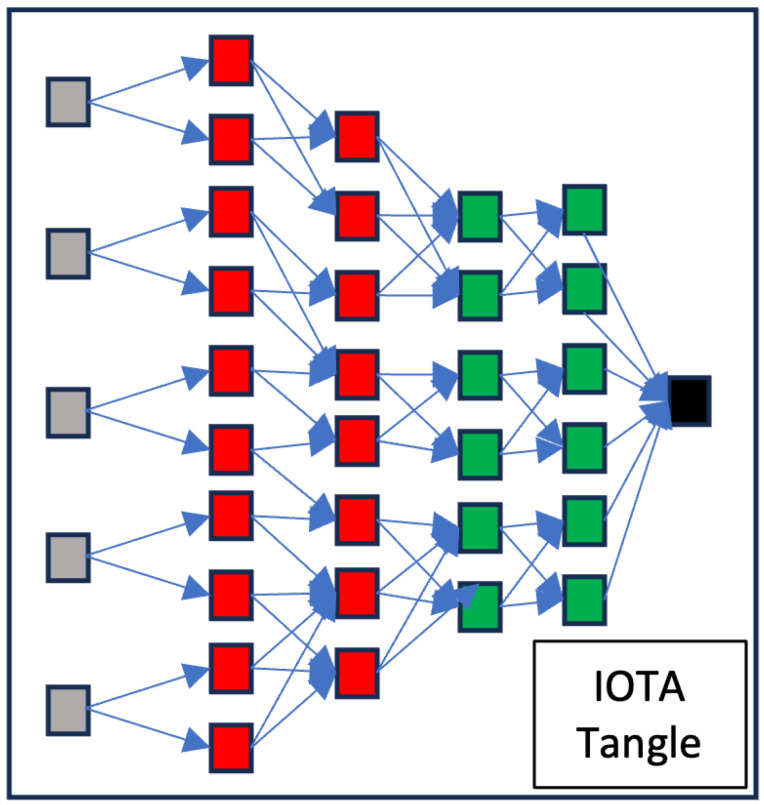
IOTA’s Tangle.

IOTA’s Tangle, a creation of the IOTA Foundation, has been recognized as a potential solution to address the limitations of traditional blockchain technology [6]. Unlike a standard blockchain, the Tangle does not consist of blocks or chains but is based on a Directed Acyclic Graph (DAG)—a structure of vertices connected by unidirectional edges that do not form loops—as shown in Figure 1. As the figure depicts, black represents the genesis; green indicates confirmed transactions; red signifies uncertain transactions (about their full acceptance); and grey denotes tips (transactions still needing validation). This design is essential for IOTA’s decentralized, distributed, immutable, and shareable digital ledger, which stores transactions efficiently. The primary goal of IOTA is to cater to the immense volume of transactions occurring within the extensive network of interconnected IoT devices, necessitating a scalable ledger system [23]. In the Tangle, each peer contributes to the network’s consistency by validating and approving two previous transactions before initiating a new one, eliminating the need for transaction fees and facilitating faster acceptance of new transactions.

IOTA employs a random tip selection strategy influenced by cumulative weights to confirm transactions. This approach is crucial in managing the network’s load, as the frequency of transactions can be modeled using a Poisson point process, with the parameter λ dictating the transaction arrival rate. A low λ indicates slower transaction rates and potential latency, highlighting the importance of balancing network activity for efficient data management.

IOTA’s system addresses the problem of ‘lazy tips’—transactions that only approve older transactions without aiding network efficiency. The network uses cumulative weights in its random walk algorithm to discourage lazy tips, thus ensuring that active transactions are more likely to be approved. For example, in a set of three tips with weights 3, 5, and 2, the algorithm prefers the transaction with weight 5. This penalty mechanism for lazy tips is balanced using a Markov chain Monte Carlo algorithm, solving the issue of unapproved tips.

In the Tangle, the selection of transactions for approval is based on a weighted walk, where transactions with lower weights are less likely to be confirmed. This system implies that transaction confirmation takes time and effort. Instead, IOTA uses ‘confirmation confidence’ to determine transaction validity, a crucial measure to prevent double-spending.

To enhance security against threats like double-spending, IOTA employs a temporary consensus mechanism called the ‘coordinator’. This mechanism, managed by the IOTA Foundation, issues milestone transactions every two minutes, instantly confirming transactions approved by these milestones. This protective measure ensures the network’s security as it grows toward full decentralization and the complete activation of the Tangle’s distributed consensus algorithm.

### 3.2. Zhang, Nakanishi, Sasabe, and Kasahara’s (ZNSK) Scheme

CP-ABE stands out in public key cryptography due to its unique implementation of a single public key, unlike conventional systems, where users have distinct public and private keys linked to various attributes [24]. In CP-ABE systems, users are issued both a master public key and individual private keys by an attribute authority, with each private key tailored to the user’s specific attributes. In the study presented in [6], a novel approach was proposed that integrates the IOTA framework with CP-ABE, employing the version described in [24]. This groundbreaking work, conducted by Zhang, Nakanishi, Sasabe, and Kasahara, primarily aims to enhance security and efficiency in data communication systems. The methodology delineated by the authors involves three core processes: access rights authorization, access rights update, and access rights verification.

In access rights authorization, an object owner assigns specific policies and access rights to a token, permitting a group of subjects to execute specific actions. Encrypted using CP-ABE, the token can only be decrypted by subjects whose private keys meet its policy criteria. Once stored on the Tangle via MAM, it becomes accessible only to authorized subjects, reducing the owner’s workload and enabling a one-to-many access control model.

In the access rights update, updating access rights involves creating and dispatching a new token through the designated MAM channel. For instance, when access rights change, the owner encrypts and uploads the updated token to the MAM channel. Furthermore, tokens can be rendered inactive by marking them as ‘INACTIVE’.

In access rights verification, subjects access resources using their tokens, incorporating an initial authentication stage to deter the use of illegitimately obtained tokens. This critical two-step verification process involves both authentication and access request phases. During authentication, an OTP (One-Time Password) ensures compliance with the token’s policy, allowing only those with matching private keys to proceed. The owner then evaluates the access request by comparing the token to its original copy, denying access if verification fails. Authentic tokens lead to an assessment of the requested actions against the token’s access rights, rejecting any action not listed as unauthorized [24].

### 3.3. Limitations of the ZNSK Scheme

In the ZNSK scheme, outlined in [6], the methodology predominantly relies on a central authority for essential system operations like setup and key generation. However, this scheme does not integrate a hierarchical organizational framework, which is instrumental for implementing hierarchical key delegation mechanisms in IoT environments. This approach, focusing primarily on pre-generated secret keys and access control, neglects in-depth exploration of key management. Additionally, its continued use of a central authority model overlooks the potential benefits of a multi-authority system, posing scalability and resilience challenges in diverse IoT settings.

Our research advances the work in [6] by developing a decentralized framework incorporating hierarchical key management and distribution. By integrating hierarchical structures, our method reduces dependence on central authorities, enhancing key management efficiency across different levels. We employ IOTA and HIBE to delegate token generation to domain-specific authorities, alleviating data owners’ workload and improving system scalability. Additionally, we introduce a robust verification method with multi-authority support and digital signatures, ensuring system integrity even if a domain authority is compromised, thus significantly enhancing IoT access control security and efficiency.

### 3.4. Hierarchical Identity-Based Encryption (HIBE)

This study concentrates on deploying the Lewko and Waters version of hierarchical identity-based encryption (HIBE) in an IoT framework to bolster access control. It distinctly highlights the unique benefits offered by this particular version of HIBE. The Lewko and Waters version’s ability to offer complete security while maintaining short ciphertexts, as detailed in [25], aligns perfectly with the needs of an IoT environment, where data efficiency and transmission speed are critical. An innovative aspect of the Lewko and Waters HIBE scheme is its elimination of tags in the dual system encryption, allowing for efficient compression of ciphertexts and the removal of potential errors associated with tag usage. Such a feature is particularly relevant in IoT applications where maintaining data integrity and minimizing overhead is paramount. Moreover, the Lewko and Waters HIBE facilitates the full rerandomization of keys upon delegation, enhancing the system’s overall security. The approach is especially beneficial in complex hierarchical structures like the IoT system in question, where multiple levels of access control are present, ranging from a broad scope at Level 0 (e.g., campus-wide access) down to more specific access at lower levels (e.g., individual labs). The system’s capability ensures that even if an encrypted token is intercepted, unauthorized parties cannot decrypt it due to the lack of appropriate hierarchical credentials. Additionally, the simplicity and efficiency of the Lewko and Waters HIBE system, stemming from its reliance on static, straightforward assumptions, make it highly practical for real-world IoT applications. Its construction in a composite order group further supports the system’s robustness, aligning well with the hierarchical nature of the proposed IoT system. The need to evaluate the efficacy of hierarchical organization at various levels—from campus buildings down to individual labs—is addressed regarding hierarchy depth and the tolerable delay time in the IoT system. In conclusion, the Lewko and Waters HIBE scheme stands out as an ideal encryption solution for the IoT system’s access control mechanism, offering a balance of security, efficiency, and hierarchical flexibility crucial for effectively managing access at various organizational levels within the IoT network.

**Figure 2:**
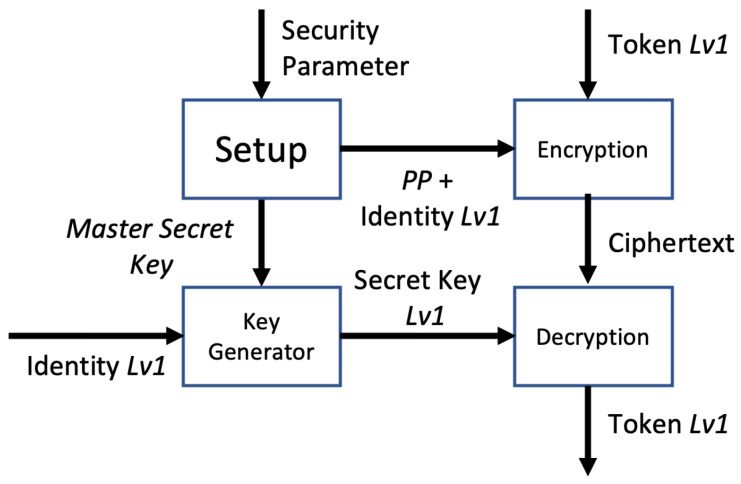
HIBE scheme for Lv0.

Four algorithms combine to form the HIBE, as shown in Figure 2:

- Setup: The central authority is responsible for executing the system setup. Retrieving the public parameter (PP) and master secret key (MSK) requires the security parameter and several properties.
- Key Generation: The secret key is generated using the MSK and a group of user identities that describe the key. For private key generation, the input is the MSK and identity set *I*; the output is secret key 1 (SK1).
- Key Delegation: SK2 is generated using SK1 and a user identity group that describes the key, which depends on the level of the organization.
- Encryption: Under the access tree topology, the token is encrypted using a combination of identities and the public parameter (PP).
- Decryption: Decryption makes use of the SK properties on each level. The token is decrypted when the set of identities satisfies the access structure.

**Figure 3:**
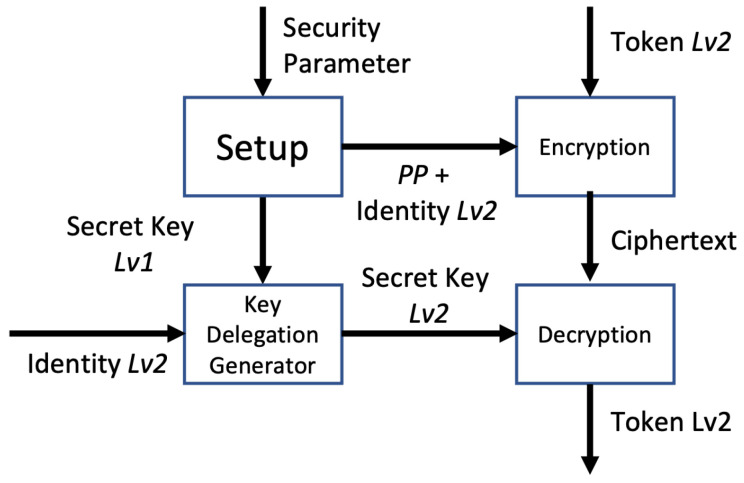
HIBE scheme for Lv1.

Figure 3 explains the HIBE scheme on Lv1. The difference between Lv0 and the other levels lies in the key delegation process. Lv0 uses the MSK, whereas the other levels use the SK.

**Figure 4:**
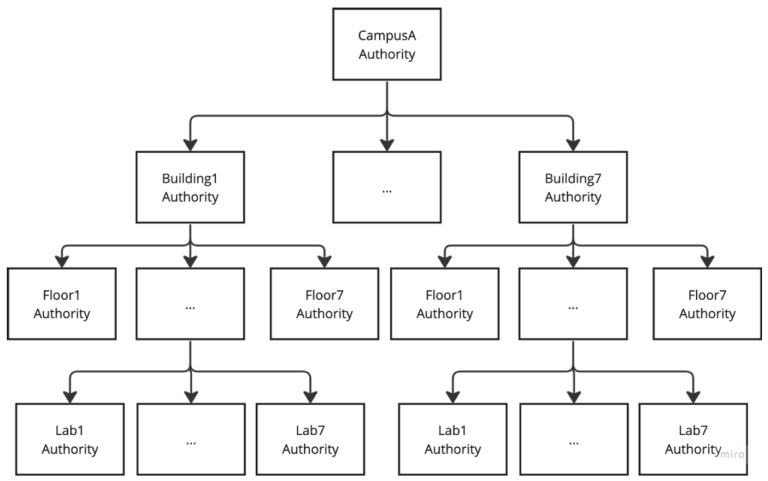
Organization tree.

For example, as shown in Figure 4, the campus authority, serving as the central authority for the hierarchy, initiates the setup process and the policies. The CA also produces the SK from its MSK and the identity of Building1, and then gives the secret key to the building authority, acting as the domain authority (DA), and entities within the Building1 domain, for example, the staff on Floor1. The building authority generates an access rights delegation process, which creates the SK for Floor1. Following this, the floor authority undertakes a similar delegation process, creating the SK for Lab1. This sequential secret key generation process continues through each hierarchical level, reaching the lowest tier, Lab1.

In the IoT, access control must be precise. For instance, even if Bob obtains keys for the entire room, if he has an app that requires access to temperature readings from a single sensor, that app should only be given the decryption key for that room. A central authority must be more scalable to provide unique fine-grained decryption keys to each person’s devices in an IoT-scale system. Furthermore, as mentioned in Section 2, such a strategy would increase security and privacy risks. Instead, Bob, who has access to the temperature measurements for the entire room, should be able to grant access to the app. A principal with access to a set of resources can typically grant another principal access to a portion of those resources.

## 4. Proposed Scheme

This section presents the innovative model for our system, meticulously designed to counter the limitations identified in Section 3.3. Central to our approach is the integration of hierarchical identity-based encryption (HIBE) with the IOTA framework, a strategic shift from centralized authority to a more distributed, hierarchical structure. This design is crucial for effective secret key distribution and managing key delegation in IoT environments.

Our system is precisely engineered to address the challenges inherent in IoT settings, focusing on scalability, resource efficiency, and adaptability. The system design significantly reduces computational demands by recognizing the resource limitations of IoT devices and data owners (DOs). In contrast to the centralized key generation presented in [6], our model delegates key functions like token generation to higher authority levels, better aligning with the needs of Industrial IoT domains. This delegation not only diminishes the computational load on DOs but also enhances the system’s scalability and flexibility, which are vital for dynamic IoT networks.

Additionally, our system employs IOTA for access control management, which is adept at handling the increasing number of devices and users while ensuring data immutability and integrity. We leverage IOTA’s distributed ledger technology to eliminate single points of failure in Industrial IoT scenarios characterized by frequent device interactions and data exchanges. We ensure data integrity and security by spreading the database across multiple IOTA nodes. The IOTA Tangle’s utilization for storing encrypted data, encrypted tokens, encrypted policies, public keys, and device serial number (DSN), coupled with its high throughput capability, makes it an ideal solution for securely managing extensive data flows in IoT systems. This comprehensive approach mitigates risks and paves the way for secure, immutable, and auditable data management in IoT networks.

### 4.1. System Model

In our proposed scheme, as delineated in this paper and distinct from the approach outlined in [6], we operate under a different set of assumptions. In [6], it was presumed that all secret keys are pre-generated before deploying the access control mechanism, thereby limiting the focus exclusively to the access control scheme itself. Conversely, our approach introduces a broader array of entities within our scheme, reflecting a more comprehensive framework. The entities in our proposed model are as follows:

1. Central Authority (CA): The CA is trusted by the whole system. The CA is responsible for registering domain authority level 1, generating the PP, MSK, and SK1, giving SK1 to level 1, generating a whole policy for the system, and storing them in the Tangle.
2. Domain Authority (DA) in each level: The DA is responsible for registering the DA, generating SK2, and giving SK2 to one level below it. The DA is responsible for an encrypted token for each level.
3. Data Owner (DO): The DO registers with the DA in its domain and requests the policy and key. The DO sends the encrypted data to the Tangle and gives the DataAddr to the DA in its domain.
4. Data Requester (DR): The DR registers with the DA in its domain. The DR can decrypt the data if its identity satisfies the access policy. The DR requests access from the DO and receives a reply with the DO’s authentication process to ensure the token is from a legitimate user.
5. IOTA: When the CA or DA generates the access policy, the encrypted token and data are stored in the Tangle.

### 4.2. Token Generation and Structure

**Figure 5:**
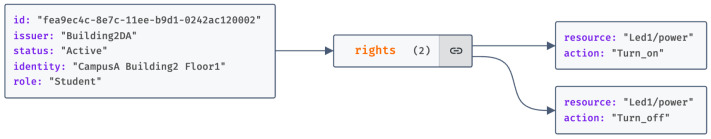
Token structure.

**Figure 6:**
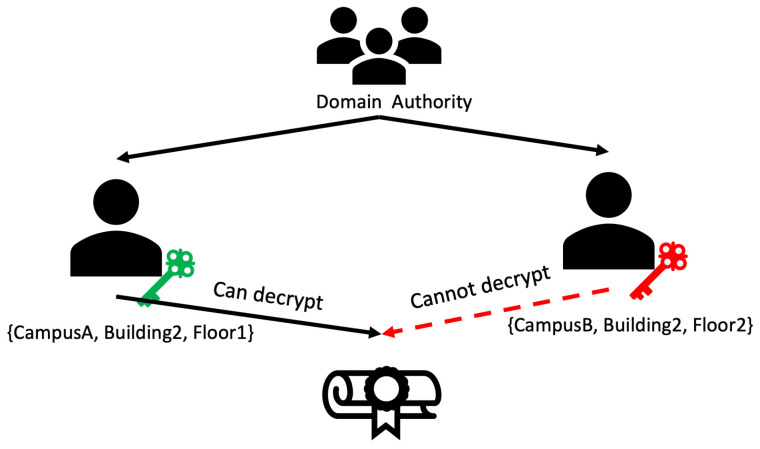
Example of access control using the IHIBE scheme.

In our proposed hierarchical access control framework tailored for IoT settings, we adopt a structure similar to the one presented in [6] for token generation. However, our methodology diverges by bifurcating policies into two distinct elements: identities and roles. For instance, as illustrated in our token structure diagram (see Figure 5), a token may be designated for the identity CampusA, Building2, Floor1. Such specificity ensures that only a DR with this identity can decrypt and effectively utilize the token (see Figure 6). Each token encapsulates several vital components, including a unique identifier, an address, the corresponding policy, and particular access privileges (e.g., adjusting lighting levels). The meticulous design of these tokens not only bolsters security by guaranteeing a precise alignment of access rights with identities but also simplifies access control management in intricate IoT ecosystems.

Initially, these roles are derived from policies generated by the CA, assuming that every IoT device within a domain adheres to the domain’s policy and does not possess autonomous rule-setting capabilities. Consequently, the CA initially generates policies for all domains within the system, creating specialized tokens for domain authority level 1 (DA Lv1).

The next step involves the CA encrypting the policy and these tokens using HIBE tailored for DA Lv1 and storing them on the IOTA Tangle. The CA then provides the addresses for these encrypted tokens (TokenAddr) and encrypted policies (PolicyAddr) to DA Lv1. In the subsequent phase, DA Lv1 downloads the encrypted policy and encrypted token from the Tangle. DA Lv1 utilizes these to derive policies for domain authority level 2 (DA Lv2) and updates the policies, subtracting its own to formulate those for DA Lv2. Following this, DA Lv1 generates tokens for DA Lv2.

Subsequently, DA Lv1 encrypts the policy and tokens using HIBE designed for DA Lv2 and stores them in the Tangle. DA Lv1 then provides the corresponding TokenAddr and PolicyAddr to DA Lv2. This token generation process from policies continues down to the lowest hierarchy level, ensuring a structured and secure approach to access management within our IoT framework.

### 4.3. Device Serial Number Generation and Structure

This section delineates the generation and structure of the Device Serial Number (DSN), a concept that was not proposed in [6]. The DSN plays a critical role in the access rights verification process, necessitating a thorough explanation of its genesis and configuration.

After registering with their respective DA, each DR receives a DSN assignment. The respective domain’s DA generates this DSN. For instance, domain authority 1 (DA1) is responsible for registering the DSN of a DR under its purview in the Tangle after the registration process.

**Figure 7:**
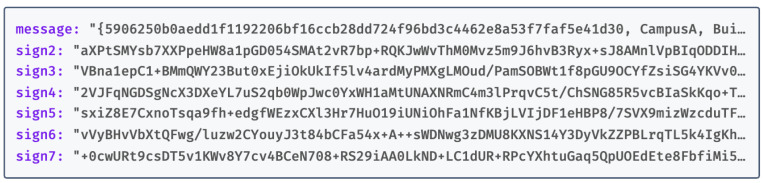
Structure of device serial number.

The structure of the DSN is JSON-formatted (see Figure 7), comprising the DSN generated by DA1, followed by signatures from DA2 to DA7. Each signature (Sign2 to Sign7) represents the validation of the DSN by the corresponding DAs, requested by DA1. For example, Sign2 is the result of DA1 requesting DA2 to sign the DSN, and similarly, Sign3 is the outcome of DA1’s request to DA3, continuing in this fashion up to DA7. DA1 then amalgamates these components, forming a JSON structure that includes the DSN and the signatures (Sign2 to Sign7).

After forming the JSON-encoded DSN, the system encrypts it using HIBE tailored to the identity of the DR. Subsequently, the system uploads the encrypted DSN to the Tangle. This structured approach lays the groundwork for an additional verification layer within our access rights verification process.

### 4.4. Access Control Mechanism

#### 4.4.1. Access Rights Delegation

In our framework, unlike the approach used in the ZNSK scheme referenced in [6], we introduce a vital component: access rights delegation. This process is indispensable for key distribution across all entities in our system. The CA or DA assigns access permissions and HIBE keys to lower hierarchy entities. Initially, the CA generates a master secret key (MSK) and issues a secret key (SK) to the domain authority at the first level (DA Lv1), laying the groundwork for the system’s trust architecture. This step is pivotal in initiating a trust chain that permits subsequent levels of DAs to delegate access rights further. Our methodical and secure approach to the access right delegation process is vital for preserving the security and efficacy of our system’s access control and security protocols across various organizational strata. Addressing the limitations in [6], our system integrates access rights delegation, overcoming the assumption of pre-generated secret keys and focusing beyond mere access control. Our methodology clarifies the processes of key generation and delegation within a hierarchical IoT environment.

**Figure 8:**
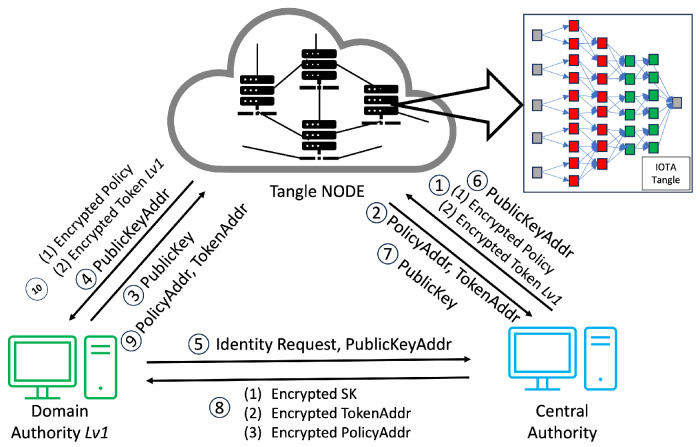
Proposed scheme: relation between CA and DA.

**Figure 9:**
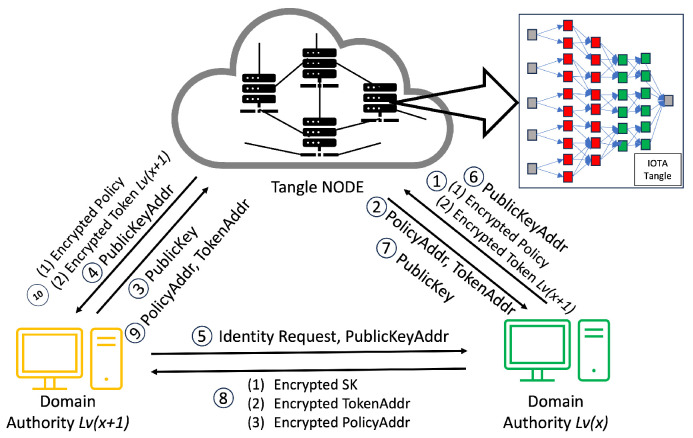
Proposed scheme: relation between DA Lv(x+1) and DA Lv(x).

**Figure 10:**
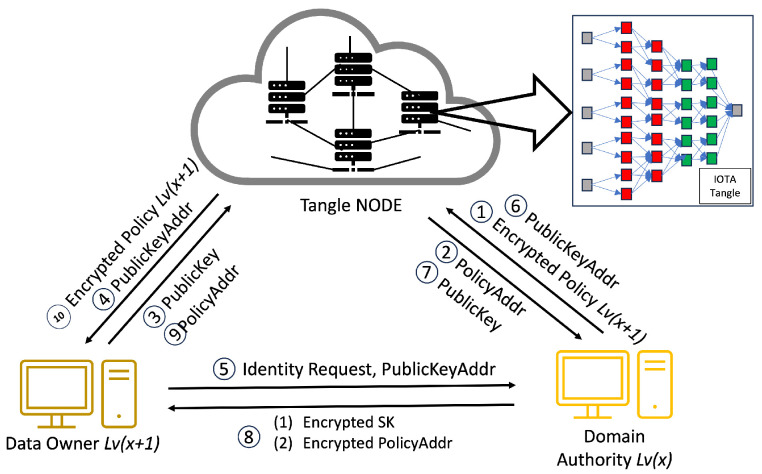
Proposed scheme: relation between DA and DO.

**Figure 11:**
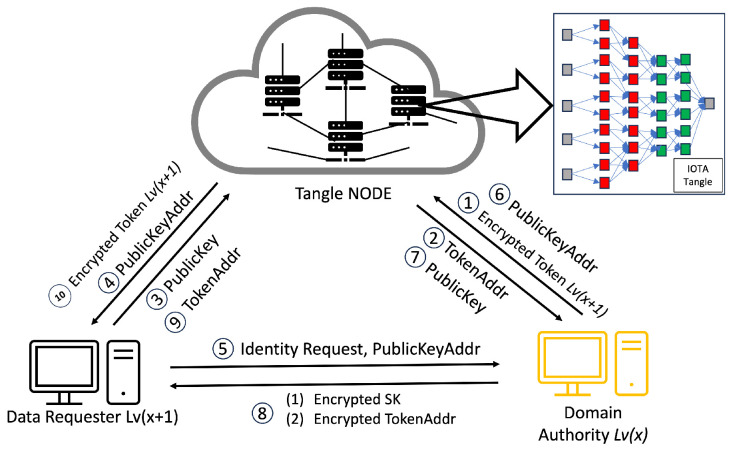
Proposed scheme: relation between DA and DR.

This procedure, executed by the CA or DA (see Figure 8, Figure 9, Figure 10 and Figure 11) entails bestowing keys on entities a tier below, such as DA Lv(x+1) and all subordinate-level entities. The CA plays a fundamental role in the hierarchical access control structure, notably during the early stages of key generation and delegation. It starts with the CA generating an MSK and creating an SK for the DA of Building1 (DA Lv1), establishing a foundational trust within the system.

**Figure 12:**
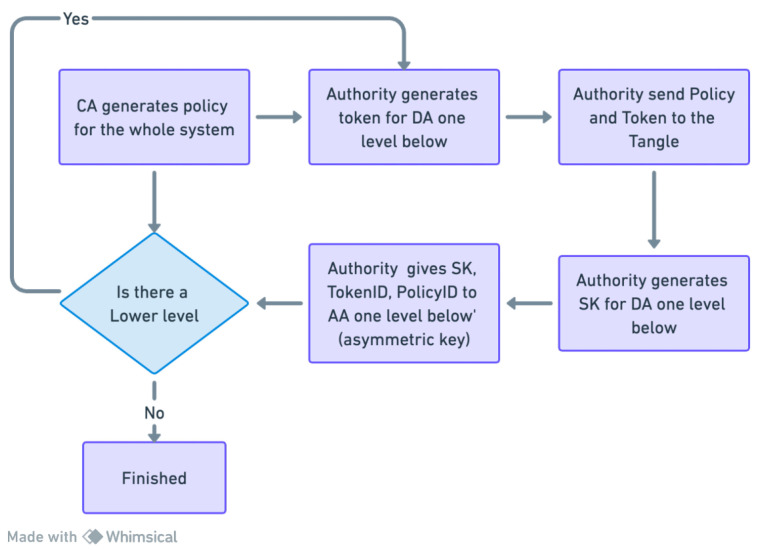
Proposed scheme: access rights delegation.

The CA utilizes asymmetric encryption to securely transmit the SK, along with the TokenAddr and PolicyAddr, to DA Lv1 (as shown in Step 8 of Figure 8). Employing the DA Lv1’s public key, stored in the Tangle, the CA encrypts the SK derived from the IHIBE scheme. Upon receipt, DA Lv1 decrypts the SK with their private key and securely archives it, completing the first key distribution level. Subsequently, DA Lv1 initiates a process of key delegation, creating a new secret key (SK2) for the DA of Floor1 (DA Lv2). By leveraging HIBE properties, this hierarchical key delegation allows entities like DA Lv2 to generate new keys for subordinate levels, such as DA Lv3 (see Figure 12). This pattern of key delegation and secure storage is replicated throughout the hierarchy, establishing a trust chain and secure key management crucial for the system’s integrity and security. The same encryption method applies to the transmission of the PolicyAddr and TokenAddr. Upon receiving the TokenAddr, each entity decrypts it to access an encrypted token from the Tangle.

#### 4.4.2. Access Rights Authorization

We modeled our access rights authorization implementation on the approach presented in [6], specifically focusing on aspects like token generation, token encryption, and uploading to the Tangle. Nonetheless, we have extensively adapted and expanded this approach to align with the complexities of a hierarchical organization in a multi-authority IoT environment. We have intricately defined the relationships of all entities with their respective domain authorities, encompassing the relationships between the CA and DA, DA Lv(x+1) and DA Lv(x), and the DA and DO, as well as the DA and DR.

Our framework’s access rights authorization process enables all entities within an organization to execute specific operations on IoT resources by policies set by a central authority. This structured approach ensures compliance with identity-based policies, defining the extent and nature of actions permissible for each entity. The allocation of unique identities to DOs and DRs, coupled with the issuance of capability tokens encapsulating these identities and their access rights, is a hallmark of our system.

Our approach assumes that IoT devices functioning within an organization are mandated to comply with the established policies of that organization. Consequently, IoT devices under the jurisdiction of a DO are prohibited from autonomously formulating policies. Instead, individuals must adhere to the policies established by the central authority, which possesses the exclusive prerogative to create and enforce policies. Furthermore, each DR must comply with these policies. To accomplish this, the DO or DR in the domain obtains identities from the authority at their corresponding levels.

In the specific area of Industrial IoT within a designated domain, adherence to policies established by a central authority eliminates the need for highly granular encryption methods. Instead, associating encryption with the locations of the DR, as exemplified by our smart campus scenario, is adequate. Consequently, with the central authority ruling the policy, we can decrease the computational burden on the DO by transferring the responsibility of token generation and uploading to the Tangle to the authority. This mechanism ensures that even a DO with limited computational power can smoothly manage the process, which is critical given the potentially high volume of tokens generated for a domain. In this context, the suitable method is Hierarchical identity-based encryption (HIBE). For example, the DRs, such as students or lecturers in {CampusA, Building1, Floor1, Lab1}, exhibit similar identities but possess different capability tokens. Based on these foundations, our system has been designed to effectively manage this complexity, thereby establishing an access control mechanism. This technique enables a differentiated access control system, wherein students can only view the data, whereas lecturers can modify the data. Consequently, this policy framework accommodates varying identities and their corresponding actions.

The fundamental principle of access rights authorization lies in the utilization of identities, such as “Student” and “Lecturer” in the context of “Building1”, as the fundamental components of access control rules. HIBE allows data to be encrypted using a set of identities, and only users with the appropriate identities can decrypt the data.

In the system we propose, Step 1 is illustrated in Figure 8, where the CA initiates the process of access rights authorization. This mechanism involves the generation of tokens and policies within the CA’s domain, with a specific focus on the interaction between the CA and DA. Firstly, the CA encrypts these tokens and policies, storing them in the Tangle. The Tangle, in return, provides the CA with the PolicyAddr and TokenAddr (Step 2 in Figure 8). A DA seeking to become part of the system is required to upload their PublicKey to the Tangle. Following this, they must submit the PublicKeyAddr provided by the Tangle to the CA (Step 3 in Figure 8). The DA then sends an identity request to the CA along with their PublicKeyAddr. The CA downloads the DA’s PublicKey using the PublicKeyAddr and, once obtained, encrypts the DA’s TokenAddr and PolicyAddr using asymmetric encryption before sending them to the DA (Steps 5–8 in Figure 8). The DA decrypts these using their private key and downloads the policy and token from the Tangle using the provided PolicyAddr and TokenAddr, receiving the encrypted policy and encrypted token from the Tangle (Steps 9–10 in Figure 8).

**Figure 13:**
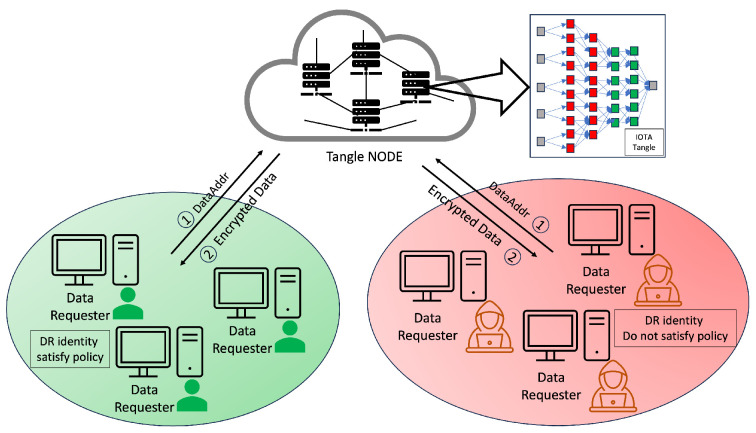
Proposed scheme: access rights authorization.

As mentioned earlier, the process mirrors the sequence for the relationship between a DA and another DA at a lower level. The case of the relationship between a DA and a DO follows a similar pattern to the DA–DA relationship but with a key difference: the DA only provides the PolicyAddr to the DO for data encryption purposes (Step 8 in Figure 10). In this process, the DO does not require immediate token possession. Instead, the token’s retrieval occurs during the verification stage. After receiving the PolicyAddr, the DO decrypts it using their private key. This decrypted PolicyAddr is then used to encrypt the data uploaded to the Tangle. Lastly, in the DA–DR relationship, the process is akin to that in the DA–DA interaction. However, the DA only provides the TokenAddr to the DR without the PolicyAddr (Step 8 in Figure 11). The DR then uses this token for the access request to the DO. After receiving the TokenAddr, the DR uses their private key to decrypt it and proceeds to download the encrypted token by submitting their TokenAddr and DSN to the Tangle to obtain the corresponding DSNAddr (illustrated in Step 9 in Figure 13). If the DR’s identity satisfies the required conditions, they can decrypt the token (Figure 11). The policy embedded in each token specifies the access rights that define which DR is permitted to carry out particular operations on the IoT device. For instance, a policy “Campus1, Building1, Floor1, Lab1” would authorize the assigned DR to perform two distinct actions—turnOn and turnOff—on a resource labeled ‘Buzzer’. After establishing the policy and corresponding access rights, the DA constructs a token adhering to a specified structure and encrypts it using HIBE in line with the policy. Consequently, only those individuals whose attributes align with the policy’s requirements can decrypt and comprehend the information. This token structure allows the DR to determine, upon satisfying the policy conditions, which devices and actions they are authorized to access and execute, thus ensuring a secure and precise allocation of access rights within the IoT environment.

#### 4.4.3. Access Rights Verification

**Figure 14:**
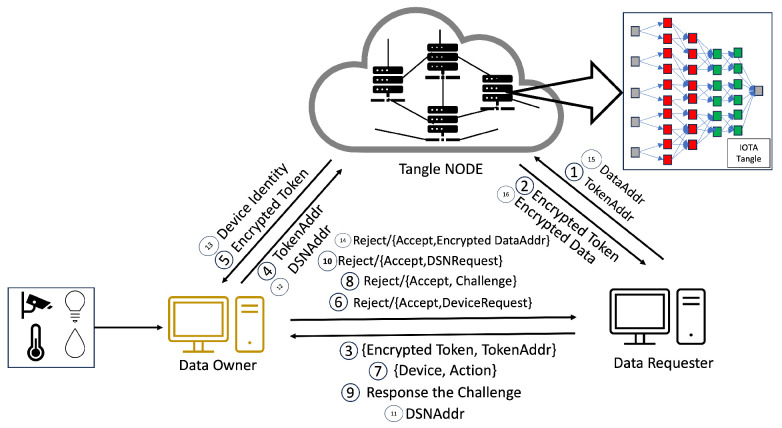
Proposed scheme: relation between DO and DR.

Our methodology, in line with the scheme in [6], involves the use of tokens by the DR for accessing resources, a process that is depicted in Figure 14. Our system introduces an enhanced verification flow, beginning with an authentication phase incorporating One-Time Passwords (OTPs) to mitigate the risk of unauthorized token usage and transfer. This initial authentication step is critical to prevent risks such as illicit token exchanges or theft by nefarious entities. Each identity in our system is assigned a unique token, enabling the detection of anomalies through the DR’s authentication.

Beyond adopting this authentication phase, our system significantly restructures the verification process to address various security challenges, including stolen tokens, stolen encrypted tokens, compromised tokens, or even compromised authority scenarios. Our approach introduces a third verification layer, device serial number verification, in addition to the conventional authentication and access request phases, where we compare the token from the data requester against the token obtained from the Tangle. This additional layer further bolsters the security of our system, ensuring a more robust and multi-dimensional verification process. By integrating this tripartite verification sequence, our system offers enhanced protection against a broader range of security threats, fortifying the integrity of the token-based access control in IoT environments.

This paper introduces a novel verification mechanism named the last-word challenge (LWC), which DA1 implements for registering a DR within the IOTA Tangle’s framework. To exemplify this mechanism, let us consider the setup of the LWC by DA Floor2 (DAF2). DAF2 issues encrypted InitiativeLWC and ReceiveLWC to DA Lab1 (DAL1). The ReceiveLWC comprises a series of words that DAL1 utilizes to request signatures from DAL2 to DAL7, whereas other DALs employ the InitiativeLWC to solicit a signature from DAL1. The transmission of these encrypted components is facilitated through asymmetric encryption, utilizing a public key derived from the IOTA Tangle.

When DAL1 intends to acquire a signature for a specific DSN from a DR, it dispatches a combination of “DSN, Identities” and the LWC to DAL2. The DSN sent by DAL1 is signed using the Elliptic Curve Digital Signature Algorithm (ECDSA) to ensure integrity and authenticity. Upon receipt, DAL2 decrypts the LWC and verifies it against its records. In the event of a match, DAL2 proceeds to append its signature to “DSN, Identities”. Subsequently, DAL2 transmits its signature, coupled with an UpdateLWC, back to DAL1. DAL1 sequentially replicates this procedure with DAL2 through DAL7. Upon securing all requisite signatures, DAL1 compiles a DSN JSON, secures it via HIBE, and proceeds to upload it to the Tangle.

**Figure 15:**
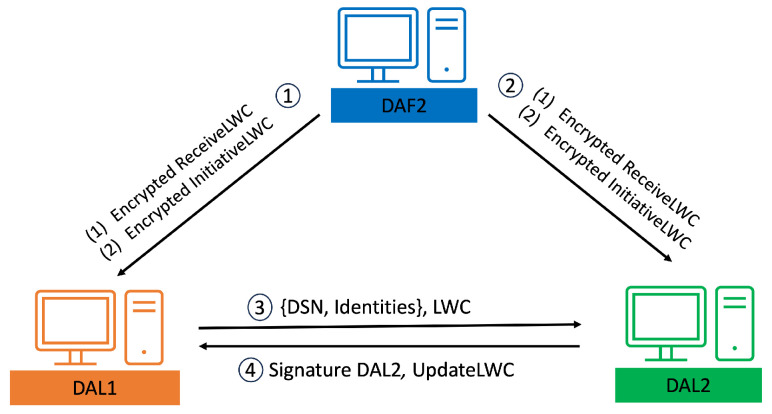
Proposed scheme: last-word challenge.

Furthermore, all public keys from DAL1 to DAL7 are stored within the Tangle, enhancing the security and accessibility of the verification process. The addresses of these public keys are provided by DAF2 concurrently with the delivery of the ReceiveLWC and InitiativeLWC. The comprehensive verification process is delineated step by step in Figure 15.

The verification process unfolds in three stages. The first stage commences with the DR presenting an encrypted token and their TokenAddr to the DO, marking the outset of the access rights verification process. In Step 6 in Figure 14, the DO verifies the token’s authenticity and integrity by comparing the DR’s encrypted token with the version retrieved from the Tangle using the TokenAddr. Success in this stage leads to Step 7, where the DR submits “Device, Action” information to the DO, enabling the DO to cross-check these details against the token’s provisions to ascertain the action’s legitimacy.

After completing Step 9 and receiving an acceptance, the process advances to Step 10, as shown in Figure 14, where the DO sends a DSN request. Subsequently, the DR transmits a DSNAddr, which the DO utilizes to download the DSN from the Tangle. The DO conducts a thorough verification of signatures from DA2 to DA7. If all signatures are validated as authentic and the DSN corresponds with the DR’s DSN, the DO then grants data access by providing an encrypted DataAdd or access to the requested device, as detailed in Step 10 in Figure 14.

## 5. Implementation

Our study presents two distinct scenarios featuring the complete set of entities (CA, DA, DO, and DR). In the first scenario, all entities are hosted on Amazon Web Services (AWS), leveraging a high-capacity Intel(R) Xeon(R) CPU E5-2686 v4 @ 2.30 GHz with 4 GB of RAM, serving as a performance benchmark in a robust, cloud-based environment. The second scenario involves implementing all entities on a Raspberry Pi 4, a device representing resource constraints, equipped with a Broadcom BCM2711, Quad-core Cortex-A72 (ARM v8) 64-bit SoC @ 1.8 GHz with 4 GB of RAM. This methodological choice is made by envisaging practical challenges in resource-limited settings, such as waste collection, where optimal route finding and real-time adaptability are essential despite resource constraints [26]. We aim to mirror these real-world conditions, exploring our system’s scalability and efficiency across vastly different operational conditions and computational resources, thus gaining crucial insights into the system’s adaptability and limitations in varied environments. In this study, we utilize charm library for the HIBE and CP-ABE, while pycryptodome library is employed for the ECDSA.

An in-depth evaluation of the system’s throughput efficiency is conducted, specifically focusing on the execution times, as discussed in the introductory part of Section 6. It is pertinent to mention that this assessment deliberately excludes measuring the communication time between entities. This exclusion is based on the significant disparity between the communication time and the Tangle upload process. As a result, the inclusion of communication time in the assessment is deemed not to provide comparable or insightful contributions to the understanding of the Tangle upload process’s efficiency, which constitutes the primary focus of this study.

Additionally, implementing all entities on a Raspberry Pi 4 is strategically chosen. This decision is predicated on the assumption that using a Raspberry Pi 4 is more economical, thereby allowing for an extended comparison with systems with higher computational power but that are also more expensive. Such an approach facilitates an exploration of the trade-off between cost and execution time. By employing a Raspberry Pi 4, which is recognized for its economic viability, the study gleans insights into the impact of cost considerations on system performance, especially concerning execution efficiency. This aspect underscores the significance of balancing economic feasibility with technical efficiency, providing a well-rounded perspective on system performance in diverse scenarios.

The execution time examination is crucial to understanding the impact of incorporating new identities and extending hierarchical levels on the adeptness of our system in handling access requests over time. Additionally, our simulation encompasses the access rights verification process, wherein we calculate the execution time for the DO to download the encrypted token and perform the decryption. We specifically exclude the measurement of communication time for access requests between the DO and the DR, as well as the time taken for the challenge-response and LWC mechanisms. Our methodology includes precisely timing these execution processes across various operational levels, providing valuable insights into the system’s efficacy in a smart campus context.

Our investigation also employs two scenarios to compare execution times across three hierarchical organizational levels within two distinct computational settings: AWS and Raspberry Pi 4. Our focus is to exclude the processes of uploading and downloading encrypted tokens, thereby concentrating on the primary operations in both AWS and Raspberry Pi 4 environments. This approach allows us to precisely evaluate and contrast the performance capabilities at these environments’ three hierarchical levels. We diligently record the execution times for each identity level in the access hierarchy, systematically repeating the process for 14 iterations to ensure a comprehensive and accurate analysis of our findings.

Next, we examine the repercussions of incrementally adding identities, starting from 10 and scaling up to 640. This part of our analysis aims to ascertain the ideal operational depth of our system. Furthermore, we aim to observe the significant impact of this identity increase on the execution time, CPU usage, and memory usage. By analyzing the execution time, we aim to deduce the maximum hierarchical depth achievable by devices with the computational power of both AWS and Raspberry Pi. Regarding CPU and memory usage, we aim to determine how the addition of identities can influence these parameters, which, in turn, can affect the workload capacity and efficiency of the devices. This comprehensive approach enables us to draw meaningful conclusions about our system’s scalability and resource management in varied computational environments.

In our updated system architecture for the IOTA Mainnet, all entities function as clients and interface with the IOTA load-balanced public Tangle endpoint, located at https://api.lb-0.h.chrysalis-devnet.iota.cafe/. This setup facilitates the operations of the DA, DO, and DR, encompassing tasks such as issuing, uploading, and downloading encrypted tokens, encrypted policies, the public keys of the entities, and their respective DSNs. Additionally, it is crucial to highlight the use of different nodes in our system’s deployment: the public Tangle is utilized to facilitate the uploading and downloading of data within the Tangle network on 23 December 2023, whereas the DSN data are uploaded using a private Tangle on 22 January 2024 via http://ec2-54-234-125-101.compute-1.amazonaws.com:14265. This integration with IOTA’s current infrastructure ensures optimized communication and efficient data management in the network, which is essential for maintaining the integrity and functionality of our IoT system in smart city applications.

In conclusion, we engage in a thorough security analysis of our system and address four key scenarios: theft of encrypted tokens, theft of tokens, compromise of both encrypted tokens and tokens, and potential compromise of the CA or DA. This extensive security evaluation is designed to robustly and clearly articulate our system’s resilience and reliability under various adversarial conditions, affirming its suitability for secure IoT environments.

As a summary of our findings on the impact of the execution time on throughput, we determine that shorter execution times correlate with better throughput performance. Since implementing Lewko and Waters’ HIBE scheme [25], which features security independent of specific hierarchy depths—unlike the paper by Boneh, Boyen, and Goh [27], where security varies with depth—we endeavor to identify the adequate hierarchy depth.

Our investigation focuses on the effects of systematically adding more identities, beginning with 10 and scaling up to 640. This effort aims to identify the upper limit of hierarchical levels that can maintain execution times within an acceptable range for the various IoT applications discussed in this paper, aligned with the tolerable time constraints outlined in [28].

## 6. Performance Evaluation

This section thoroughly evaluates our scheme’s throughput performance, focusing on execution time. This evaluation is crucial for understanding how adding identities and deepening hierarchical levels can impact the system’s ability to process access requests efficiently over time. We conduct this analysis by meticulously measuring the execution time for each operational level, thereby assessing the system’s functionality in a smart campus context.

Initially, we examine the execution times at various hierarchical levels, comparing these metrics between two different computational environments: AWS with specified computational capabilities and a Raspberry Pi 4. This comparison is vital to determine the effective hierarchical depth for systems where the DO and DR operate on computers without resource constraints and those running on Raspberry Pi 4. To achieve an accurate assessment, we sample execution times for each identity level in the access tree, performing 14 iterations at each level.

Subsequently, we explore the impact of adding identities in an exponential growth pattern, starting from 10 and extending up to 640 identities. This aspect of our analysis is geared toward determining the optimal operational depth of our system. Based on the minimal variances observed in our initial execution time measurements—with access rights delegation averaging around 0.000916 s, access rights authorization (excluding Tangle upload) averaging approximately 0.01874 s, and access rights verification (excluding Tangle data download) averaging nearly $1.124\times 10^{-6}$ s—we opted to sample each operation once for this evaluation phase.

Finally, we delve into a detailed security analysis of our system, addressing four critical scenarios: theft of encrypted tokens, theft of tokens, compromise of both encrypted tokens and tokens, and potential compromise of the CA or DA. This comprehensive security assessment aims to provide a robust and clear understanding of our system’s resilience and reliability under various adversarial conditions, ensuring its suitability for secure IoT environments.

### 6.1. Access Rights Delegation

**Figure 16:**
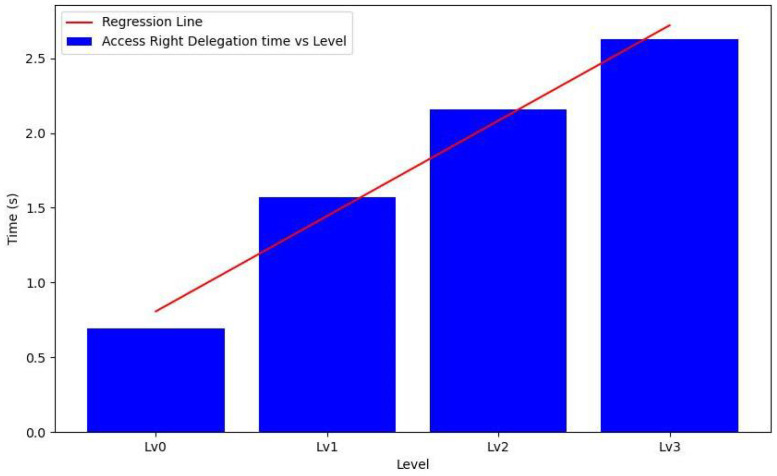
Execution times on AWS for access rights delegation across hierarchical levels: Lv0 to Lv3.

Figure 16 depicts a mathematical relationship illustrated by the regression line equation, derived from a linear fit to the data points. This equation clarifies how time varies with different levels, ranging from Lv0 to Lv3. In this equation, represented as y = mx + c, ‘m’ represents the slope, and ‘c’ is the y-intercept.

The linear regression analysis was conducted to quantify the relationship between the number of identities in a system and the corresponding time required for access rights delegation. The computational times recorded at different hierarchical levels, ranging from Lv0 to Lv3, were subjected to statistical analysis to ascertain the nature of this relationship.

The linear regression results yielded a slope (β) of approximately 0.6381 s/level, suggesting that each additional identity level introduced an average increase of 0.6381 s in the delegation time. The regression equation’s intercept (α), representing the delegation time for the base level (Lv0), was found to be approximately 0.8066 s.

A high coefficient of determination ($R^{2}$) of 0.9789 was observed, indicating that the linear model could explain 97.89% of the delegation time variance. This high $R^{2}$ value indicated a robust linear association between the identity levels and the delegation times, implying a consistent and predictable increment as the system expanded in complexity. Furthermore, the *p*-value was 0.0106, significantly below the conventional significance level (α) of 0.05. This value indicated that the correlation between the levels and delegation times was statistically significant, validating the reliability of the linear predictive model. The standard error of the regression slope was approximately 0.0662 s, indicating high precision in the slope estimation and, by extension, the predicted increase in delegation time per level.

The analysis confirmed a near-linear growth in the delegation time with each added identity, which is critical for anticipating system behavior in larger-scale deployments within IoT environments. The practical ramifications for system design are substantial, allowing for informed decision making regarding resource allocation, system architecture, and anticipated performance thresholds.

**Figure 17:**
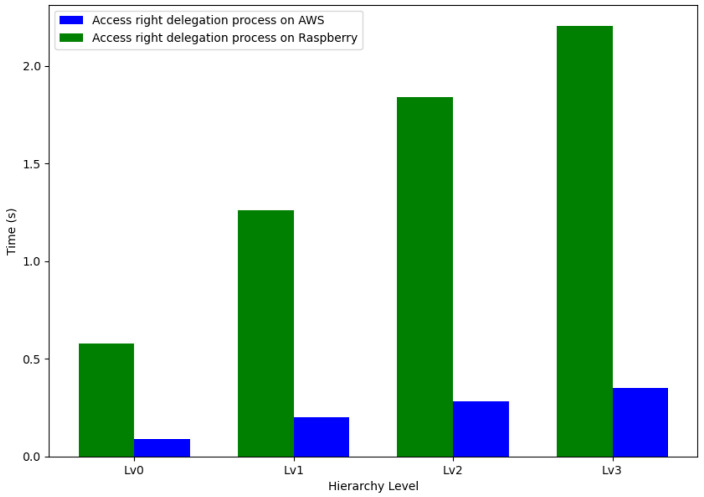
Execution times for access rights delegation: AWS vs. Raspberry Pi 4.

Our system, which focuses on a hierarchical structure, was specifically designed to suit IoT environments that encounter resource limitations (Figure 17). It adopts a hierarchical key management approach, where key generation and delegation are seamlessly managed from upper to lower levels within the hierarchy. This structure enables higher levels to decrypt messages from lower levels using encrypted tokens. Crucially, by integrating these encrypted tokens, encrypted policies, public keys of entities, and DSNs of entities into the IOTA framework, our system not only guarantees the immutability, integrity, and high availability of data across the IOTA network’s nodes but also significantly reduces the burden on data owners. This reduction is achieved by transferring the responsibilities of token generation, encryption, and storage in the Tangle to the authoritative levels, thereby minimizing the tasks traditionally associated with data owners.

Furthermore, this approach markedly enhances scalability by circumventing the need for a centralized database, leading to high transaction throughput and enabling many concurrent transactions. This hierarchical model’s design is instrumental in ensuring effective and secure access control in resource-limited IoT environments and plays a pivotal role in streamlining operational efficiencies by reassigning critical tasks to more capable hierarchical layers. As a result, our system represents a significant advancement in managing access control complexities while maintaining performance and security standards in IoT environments.

### 6.2. Access Rights Authorization

**Figure 18:**
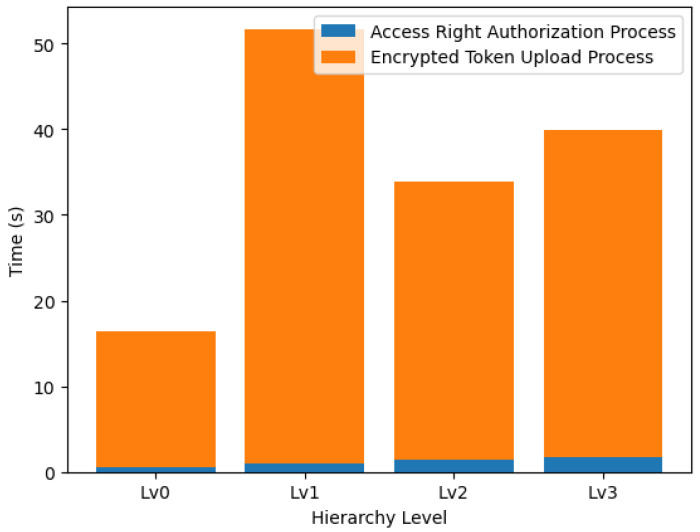
Execution times on AWS for access rights authorization across hierarchical levels: Lv0 to Lv3.

Figure 18 presents a comparative overview of the computational costs associated with two critical processes: the encryption of data (denoted in blue) and the subsequent upload of these data to the Tangle. The system’s performance was evaluated by measuring the time taken for two critical processes: access rights authorization and encrypted token upload. These metrics were recorded across a spectrum of hierarchy levels, namely Lv0, Lv1, Lv2, and Lv3, representing increasing complexity levels within the system’s structure.

The encryption times, represented in blue, gradually increased as the hierarchy level ascended. Starting at 0.4949 s for Lv0, the time required stretched to 1.7942 s by Lv3. This trend suggests a linear relationship indicative of the additional computational overhead introduced by each successive level.

Conversely, the upload times, represented in orange, exhibited a more variable pattern. While Lv1 demonstrated a significant spike at 50.6432 s, Lv2 and Lv3 reflected a decrease, with times of 32.3413 and 38.1509 s, respectively. This non-monotonic behavior could be attributed to network fluctuations or varying system loads during uploading.

The combined stack bars in the generated chart elucidate the cumulative time impact of both processes at each level. The access rights authorization process constitutes a relatively constant fraction of the total time. In contrast, the encrypted token upload process is more erratic, suggesting that the latter was the primary driver of time variability across levels.

To understand the performance implications of system hierarchy on encryption processes, we measured the time taken to complete access rights authorization on two different platforms: AWS and Raspberry Pi 4. The analysis was carried out across four levels of the system hierarchy, Lv0 to Lv3.

The Raspberry Pi 4 exhibited a significantly higher encryption time at each hierarchy level compared to AWS. Specifically, the encryption process on the Raspberry Pi 4 took 260.89%, 264.14%, 267.85%, and 257.19% more time compared to AWS at levels Lv0, Lv1, Lv2, and Lv3, respectively. These percentages underscore a substantial difference in performance between the two platforms, with the Raspberry Pi 4 consistently taking over 2.5 times longer compared to AWS to complete the same encryption task.

This pronounced discrepancy can be attributed to the differences in computing power and operational environments between the cloud-based AWS and the hardware-limited Raspberry Pi 4. Despite the increased computational resources available on the Raspberry Pi, the overhead associated with its constraint environment may have contributed to the longer authorization times observed.

**Figure 19:**
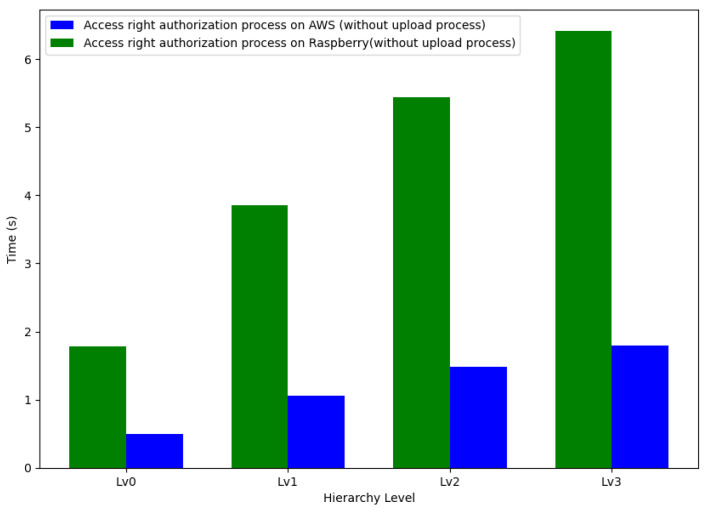
Execution times for access rights authorization: AWS vs. Raspberry Pi 4.

Figure 19 illustrates a comparative analysis of the encryption times, visually representing the performance on both AWS and the Raspberry Pi 4 across the hierarchical levels.

The results emphasize the importance of enhancing optimization and fine-tuning performance, which is particularly crucial for implementing access rights authorization in cloud-based IoT settings. Securely storing encrypted tokens within the IOTA framework ensures their availability and integrity, further reinforcing the system’s robustness. The data gathered points to a critical need for refining the upload process, a necessity that grows with the system’s expansion. While the steady growth in authorization time is both foreseeable and controllable, the inconsistency in upload times presents considerable obstacles in practical deployment situations, which could significantly affect the overall efficacy and dependability of the system.

### 6.3. Access Rights Verification

The system performance evaluation was extended to assess the decryption and download times essential for the access rights verification process. This analysis was conducted across various hierarchy levels (Lv0 through Lv3), representing incremental stages within the system’s architecture.

At the foundational level, Lv0, the decryption time was observed to be 0.0901 s, with the encrypted token download process requiring an additional 0.2500 s. As the hierarchy level increased, a consistent rise in decryption time was noted, culminating at 0.3498 s for Lv3. In contrast, the download times after decryption displayed an inverse relationship, with an initial increase at Lv1 followed by a decrease, stabilizing at around 0.167 s for higher levels.

**Figure 20:**
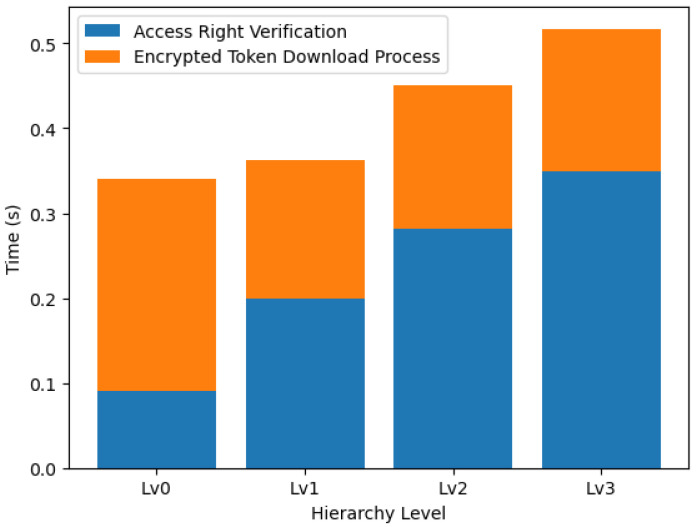
Execution times on AWS for access rights verification across hierarchical levels: Lv0 to Lv3.

Figure 20 illustrates a stacked representation of the two processes, with the bottom segment in blue indicating the decryption time and the top segment in orange representing download time. A noteworthy observation was that although the decryption time exhibited a linear increase with each level, the download time did not follow a simple linear trend, suggesting that factors other than hierarchy level complexity may have influenced it.

Regarding relative performance, the decryption process accounted for a significant portion of the total time at the lower hierarchy levels but became proportionally less dominant as the hierarchy level increased. Although the download process varied, it did not consistently increase alongside the hierarchy levels, implying a degree of variability, possibly induced by network or server performance issues.

Future investigations should dissect the underlying causes of the observed trends, specifically the variability in download times. It is essential to determine whether these are attributable to the network conditions, server response times, or other external factors. Such insights would be crucial for optimizing the system’s overall efficiency, ensuring that the access rights verification process remains swift and reliable as the system scales.

**Figure 21:**
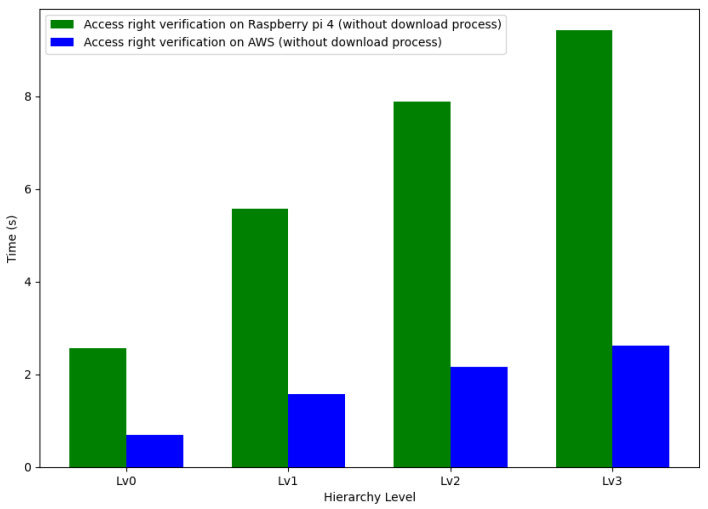
Execution times for access rights verification: AWS vs. Raspberry Pi 4.

The investigation was extended to examine the differences in the time taken to generate session keys at various hierarchical levels, contrasting the computational performance on AWS with that on the Raspberry Pi 4. This analysis covered four levels, designated as Lv0 to Lv3, each signifying a progressive increase in system complexity. Figure 21 provides a detailed visualization.

The session key generation time on the Raspberry Pi 4 was substantially higher than that on AWS across all hierarchy levels. Specifically, the generation process on the Raspberry Pi 4 required 267.60%, 254.46%, 264.62%, and 258.51% more time compared to AWS at levels Lv0, Lv1, Lv2, and Lv3, respectively. These percentages represent a marked difference in performance, with the Raspberry Pi 4 consistently taking more than two and a half times longer than AWS for session key generation.

This significant contrast in performance can be ascribed to the differing computational resources and execution environments of the cloud-based AWS service and the more constrained Raspberry Pi 4 hardware. The additional computational overhead associated with the Raspberry Pi 4’s hardware may have contributed to the increased times observed.

The results emphasize the necessity for optimization, particularly within edge computing environments, to bridge the latency gap between cloud services and edge devices. Ensuring efficient and rapid session key generation is crucial, as it is pivotal in hierarchical systems’ overall security and performance. Future efforts should focus on enhancing the efficiency of cryptographic operations in edge computing platforms, making them more competitive with cloud-based solutions.

Our implementation included a performance analysis of the last-word challenge, focusing on using digital signatures, specifically the ECDSA algorithm, in a DSN. The primary objective was to evaluate the execution time for digital signature generation and its implications on DA efficiency. The research revealed that multiple domain authorities, ranging from DA2 to DA7, were individually signing DSN data. The mean signing time was calculated to be approximately 0.0016 s, and the average verification time for each signature was found to be around 0.0024 s.

In addition to signature generation, this study assessed the time taken for DA1 to aggregate these signatures into a JSON format and the subsequent upload time to the Tangle. The average upload time, determined over seven trials, was observed to be 123.48 s, which is approximately 2.06 min. This duration formed a significant portion of the overall process time.

After delineating our system architecture, a comprehensive comparative analysis was conducted, focusing on the performance of access rights authorization relative to the operations of our system. In this analysis, particular emphasis was placed on the encryption times and upload speeds associated with access rights authorization. Variability in encryption times was documented, with values such as 0.4949, 1.0601, 1.4778, and 1.7942 s recorded. It was observed that the upload times to the public Tangle for access rights authorization were significantly faster, taking 15.9168, 80.6432, 74.9884, and 38.1509 s.

The notable disparity in upload times can be ascribed to the utilization of distinct nodes for disparate functions within the system. As previously outlined, the public Tangle was employed for access rights authorization, whereas the DSN data utilized a private Tangle for uploads. This variation in node selection, serving different purposes within the same architectural framework, reflects the system’s responsiveness to diverse network conditions, including fluctuations in network traffic, node performance, or other environmental factors that might influence the Tangle’s performance over time. This methodology is essential for ensuring the adaptability and efficiency of the system, particularly pertinent in the context of smart city applications, as it allows for a nuanced understanding and application of technological resources to meet varying operational demands.

In conclusion, this study highlights the efficiency of digital signature generation in a DSN context, demonstrating that the time required for signing and verification by domain authorities is minimal. However, the primary time consumption is attributed to compiling signatures into a JSON format and uploading them to the Tangle. The findings indicate that node and network conditions can significantly impact the overall time efficiency.

### 6.4. Evaluating the Efficiency of Access Control Mechanisms in Smart Systems: A Comparative Study on AWS and Raspberry Pi 4 Platforms

In this research, we analyzed the execution times for three critical processes in IoT systems, namely access rights delegation, access rights authorization, and access rights verification, by comparing their performance on AWS and a Raspberry Pi 4. This study aimed to evaluate the scalability and efficiency of these processes in the context of smart city applications [28] by measuring execution times across various policy settings (10, 20, 40, 80, 160, 320, and 640 identities).

**Figure 22:**
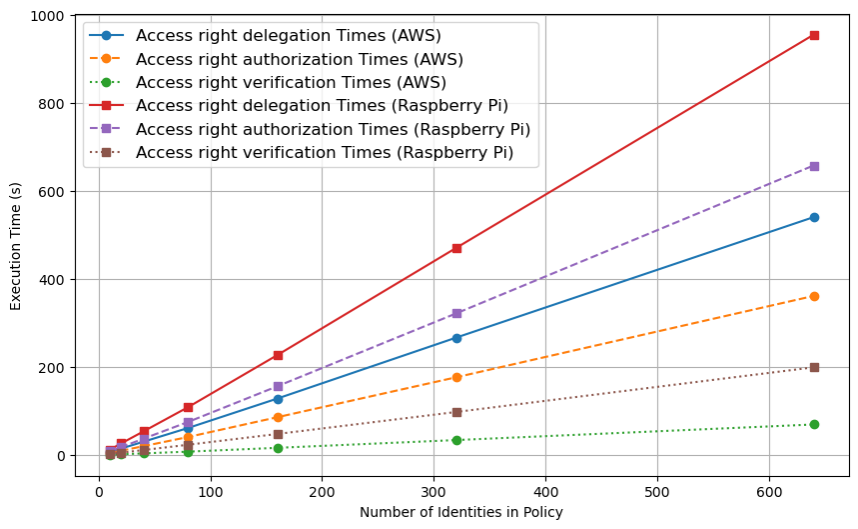
Execution times on AWS and Raspberry Pi 4.

The data, as illustrated in Figure 22, revealed a marked increase in the execution time for access rights delegation on the Raspberry Pi 4, which escalated from 12.54 s with 10 identities to 954.93 s with 640 identities. Considering this, alongside an additional average time of approximately 2 min for uploading data to the Tangle, a comparison of the tolerable delays became essential. This comparison was instrumental in determining the optimal depth on AWS and the Raspberry Pi and is presented in Table 1. While access rights authorization also exhibited increased times, albeit with slightly better efficiency compared to delegation, the increase in execution times for access rights verification was comparatively moderate, indicating it was less demanding on resources and more scalable. In contrast, the performance patterns on AWS differed significantly, highlighting the impact of platform choice on IoT system architecture.

The findings, as detailed in Table 1, reveal that the ideal number of identities in policies for smart city services is highly dependent on the chosen platform. Services like structural health monitoring and smart lighting, which are less time-sensitive, can be efficiently managed by the Raspberry Pi 4 with policy sizes of up to 640 identities. However, for more time-critical services such as waste management and traffic congestion management, along with other services with a 5 min tolerable delay, the Raspberry Pi 4 is optimally suited for policy sizes of up to 160 identities. This thorough analysis, encompassing execution times and durations for encrypted token uploads, emphasizes the crucial significance of execution time and platform choice in designing IoT systems for smart cities. These insights provide valuable guidelines for determining optimal policy sizes to meet the diverse requirements of smart city applications, supported by data from smart city service research [28].

**Figure 23:**
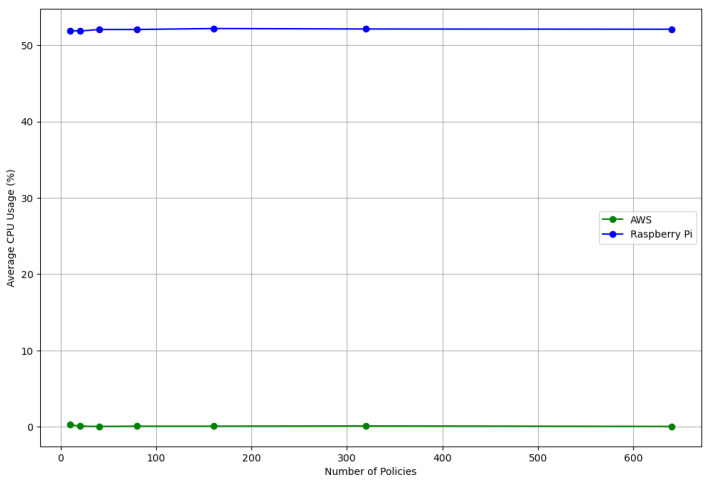
Average CPU usage vs. number of policies on AWS and Raspberry Pi 4.

**Figure 24:**
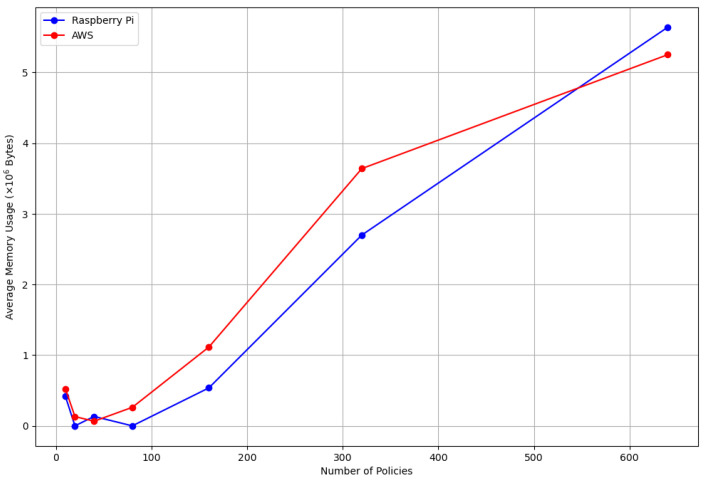
Average memory usage vs. number of policies on AWS and Raspberry Pi 4.

In the performance analysis of our IoT system on the AWS and Raspberry Pi platforms, we concentrated on two critical metrics: CPU usage and memory consumption, as depicted in Figure 23 and Figure 24. These metrics are essential for understanding IoT systems’ efficiency and resource utilization, particularly in smart environments where resource constraints are a primary concern.

The CPU usage trends observed between AWS and the Raspberry Pi across various policy sizes (ranging from 10 to 640) reveal distinct operational characteristics. The Raspberry Pi demonstrated a consistent CPU usage pattern, averaging around 52% across all policy sizes. This consistency suggests a stable load on the Raspberry Pi’s processor, albeit relatively high, which could impact other concurrent tasks or the overall system responsiveness in real-world IoT settings. On the other hand, AWS exhibited minimal CPU usage for most policy sizes, with a notable exception at the highest policy size (640), with a significant spike to 33%. This spike may indicate a threshold at which the AWS configuration begins to encounter a substantial processing load. However, AWS’s generally low CPU usage indicates its efficient handling of processing demands, likely attributed to its superior computational resources.

The memory consumption analysis further complemented our understanding of the system’s scalability and resource demands. On the Raspberry Pi, we observed an increasing trend in memory usage with the rising number of policies, starting from approximately 270 K bytes at the smallest policy size and escalating to nearly 10 M bytes at the largest policy size. This increasing memory demand highlights the challenges in scaling the system on resource-constrained devices like the Raspberry Pi. In contrast, AWS showed a similar increasing trend but began and ended at higher values than the Raspberry Pi, starting at around 500 K bytes and reaching approximately 10 M bytes. While this also signifies rising memory demands with scaling, AWS’s robust memory capabilities can likely accommodate these demands more comfortably.

The findings of this study are pivotal for IoT systems, particularly in smart environments, where the balance between performance efficiency and resource utilization is crucial. The steady, high CPU usage and increasing memory demands on the Raspberry Pi underscore the importance of optimization for both processing and memory utilization to ensure seamless operation, especially in complex or large-scale IoT deployments. Conversely, AWS, with its lower CPU usage and ability to handle increased memory requirements, offers a robust platform for more demanding applications. However, attention must be paid to potential performance bottlenecks, as indicated by the CPU usage spike at the largest policy size in extensive deployments.

In conclusion, this performance analysis provides a comprehensive overview of the system’s resource demands and efficiency, crucial for decision making regarding platform choice, system design, and scalability in various IoT environments, from smart campuses to urban IoT deployments. The contrasting performance profiles of AWS and the Raspberry Pi highlight the need for careful consideration of the operational context and resource constraints when deploying IoT systems in smart environments.

### 6.5. Comparison with the ZNSK Scheme

In this analysis, we contrast our approach with the ZNSK and IHIBE schemes, emphasizing the decryption groups guided by policies such as “CampusA”, “CampusA, Building1”, “CampusA, Building1, Floor1”, and “CampusA, Building1, Floor1, Lab1”. These policies form the identities in IHIBE scheme and attributes in ZNSK scheme and are used as information for generating the key. Contrasting with the scenarios presented in Section 6.1, Section 6.2 and Section 6.3, where DA is tasked with key generation process for their domains, this section highlights the role of the CA (Level 0) for this responsibility. The operations assessed include the setup time, the access rights delegation, access right authorization, and access right verification times. The aim was to evaluate the efficiency of both schemes in executing these critical operations.

**Figure 25:**
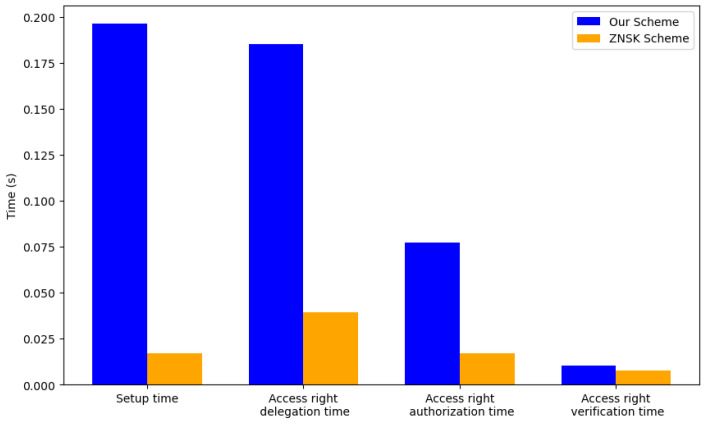
Comparison of execution times: our scheme vs. ZNSK scheme.

As illustrated in Figure 25, our scheme displayed a marked difference in operational times compared to the ZNSK scheme. The percentage differences in the operational times for the setup time, access rights delegation, access rights authorization, and access rights verification were 1048%, 369.72%, 355.57%, and 33.35%, respectively. Despite these high percentages, it is crucial to note that the absolute differences in the operational times were relatively small, being in the order of tenths of a second. Specifically, for the setup time, the variance was approximately 0.179 s; for access rights delegation, it was around 0.146 s; for access rights authorization, it was 0.061 s; and for access rights verification, it was nearly 0.0026 s. While notable in percentage terms, these minor variances are still well within the delay tolerance thresholds outlined in [28], suggesting comparable performance levels.

While our scheme showed a substantial increase in time for the first three operations, the verification time difference was relatively minor, at 33.35%. This result suggests that although significant operational efficiency disparities existed in certain areas, the impact on verification time was minimal. In practical terms, this could imply that the increased setup, access rights delegation, and access rights authorization times might be acceptable in scenarios where verification time is the primary concern.

In conclusion, our scheme exhibits longer operational times for setup, access right delegation, and access right authorization processes; compared to the ZNSK scheme, the actual time is smaller (in the order of tenths of a second) than the average tangle upload time (34.26 s) for all levels shown in Figure 18. Our scheme and ZNSK scheme require communication with the tangle in this amount of time. Moreover, our system is specifically tailored to meet the requirements of hierarchical organizations in an IoT environment, adding relevance to our findings. Future work should focus on optimizing the more time-intensive aspects of our scheme, mainly aiming to reduce setup, delegation, and authorization times to enhance its overall efficiency.

### 6.6. Security Evaluation

This section discusses a security evaluation of our system, addressing four critical scenarios: encrypted token theft, token theft, compromise of authority, and the effectiveness of the challenge-response mechanism.

#### 6.6.1. Token Theft

In this intricate scenario of IoT security, we delved into the ramifications of a DR adversary successfully stealing a token, leading to a potential breach in the system’s security. Having illicitly acquired the token, the adversary gained insight into its contents. This unauthorized access to the token initially threatened the system’s integrity. The adversarial DR, emboldened by this breach, exploited this advantage by directly submitting the stolen token to the DO.

**Figure 26:**
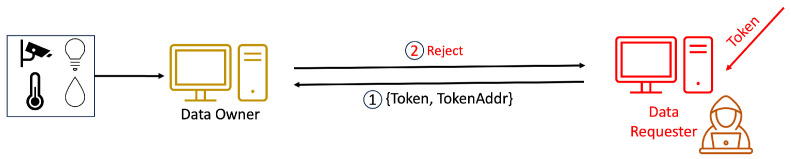
Security response to token theft scenario.

This scenario, as depicted in Figure 26, illustrated the vulnerability of tokens to theft and the strength of the IoT system’s countermeasures. The system’s security protocols were immediately activated upon the DO’s receipt of the EncryptedToken and TokenAddr. The DO examined the submitted EncryptedToken and TokenAddr as part of a rigorous verification process. This examination was crucial because the system explicitly demanded an encrypted token, yet a discrepancy arose if the DR provided an improperly encrypted token. When the DR encrypted the token incorrectly, the DO could not decrypt it, leading to the inevitable rejection of the access request. Similarly, if the DR adversary failed to provide the correct TokenAddr, the DO used the incorrect TokenAddr to download the token, resulting in an inability to retrieve the appropriate token. This series of checks and balances ensured that any attempt by an unauthorized entity to gain access was swiftly identified and neutralized, whether through masquerading as a legitimate DR or by other means. The system’s robustness in thwarting such security breaches was highlighted by its prompt and effective response in discerning and denying unauthorized access attempts.

#### 6.6.2. Encrypted Token Theft

**Figure 27:**
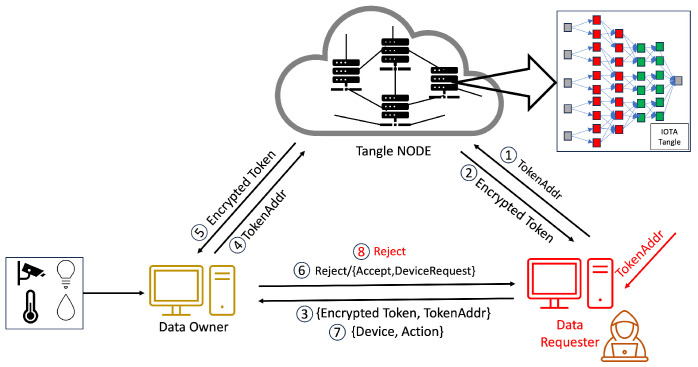
Security response to encrypted token theft scenario.

In our IoT system security scenario, we critically analyzed the theft of an encrypted token during a man-in-the-middle (MITM) attack. The DR, acting as an adversary, initially intercepted the TokenAddr from the Tangle through an MITM attack and gained knowledge of the Tangle’s address. Utilizing this information, as shown in Figure 27, the adversarial DR retrieved the corresponding encrypted token by submitting the stolen TokenAddr. However, this posed a significant verification challenge, as the adversary needed the legitimate TokenAddr corresponding to the stolen encrypted token.

In the verification process, the DO played a critical role. Upon receiving the TokenAddr from the DR, the DO queried the Tangle to download the original encrypted token. As this token was valid, it matched the one provided by the DR, thereby passing the initial verification stage.

Subsequently, the DO requested the DR to specify the target device and the intended action. This step was crucial, as the encrypted token, which contained the necessary information about the permissible device–action pair, remained undeciphered by the DR. The DR’s inability to decrypt the token rendered them incapable of correctly identifying the specific device and action authorized by the token. Due to the DR’s lack of access to the correct decryption information, this mismatch led to the DO rejecting the access request. Such a robust verification mechanism ensured that even if the initial stages were passed, any unauthorized attempt by the DR to access the system was effectively identified and prevented, maintaining the system’s security integrity.

This security scenario demonstrated the resilience of the IoT system’s security mechanisms, particularly in safeguarding against the risks associated with encrypted token theft. The system’s robust protocols ensured that unauthorized access was effectively prevented, maintaining the integrity and confidentiality of the IoT environment.

#### 6.6.3. Dual Theft of Token and Encrypted Token

The scenario commenced with a DR adversary cunningly positioning themselves as a man in the middle, surreptitiously obtaining a TokenAddr from the Tangle and discerning the Tangle’s address. This initial step began a complex chain of unauthorized actions aimed at compromising the IoT system’s security. Armed with the stolen TokenAddr, the adversarial DR retrieved the corresponding encrypted token. Next, displaying deceptive accuracy, the adversary submitted both the encrypted token and the TokenAddr to the DO, successfully passing the first layer of security validation.

The DO, adhering to protocol, forwarded the received TokenAddr back to the Tangle for verification. The Tangle responded by providing the DO with the original encrypted token, enabling the DO to perform a crucial comparison to validate the authenticity of the submitted token. Should there have been a mismatch between the two encrypted tokens, the system would have immediately rejected the access request, safeguarding its integrity. However, in this scenario, the adversarial DR’s meticulous execution resulted in a match, compelling the DO to initially accept the request and proceed to the subsequent security checkpoint.

At this juncture, despite being unable to decrypt the encrypted token, the adversarial DR knew the appropriate device and action from the stolen token. This information enabled the adversary to pass the device and action request, misleading the DO into issuing a challenge to verify the legitimacy of the requesting entity. In responding to this challenge, the adversary’s facade began to crumble. Without the proper decryption key to decipher the challenge, the adversarial DR’s response was invariably incorrect.

**Figure 28:**
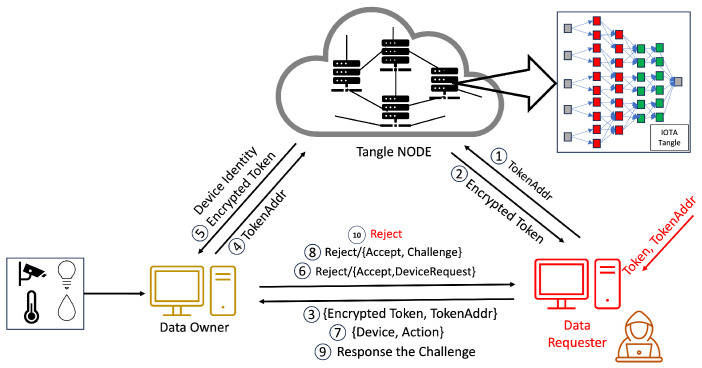
Security response to encrypted token and token theft scenario.

Upon receiving an incorrect response, the DO promptly identified the discrepancy, conclusively proving the illegitimacy of the access request. The system’s robust security architecture, as depicted in Figure 28, thus thwarted the adversary’s attempts at unauthorized access. This layered defense mechanism, incorporating token validation, device and action verification, and a decisive challenge-response protocol, collectively formed an impregnable barrier against such sophisticated attacks, ensuring the persistent security and reliability of the IoT system.

#### 6.6.4. Compromise of Authority

A critical security challenge arose when an IoT access control hierarchy authority was compromised. In systems utilizing IHIBE, where authority was charged with generating and delegating HIBE keys, the term “compromise” acquired a specific connotation. A compromise in this context referred to any breach or undermining of the security protocols that governed the generation, delegation, and management of these encryption keys. The severity of the breach depended on the level of the compromised authority. For instance, if a DA for a domain such as “CampusA, Building2, Floor2” was compromised, the breach was confined to that domain and its hierarchical subdomains. However, a compromise at the CA level signified a catastrophic breach, rendering the entire system vulnerable.

In such a scenario, the compromised authority could have distributed HIBE keys to an adversarial DR. This breach could allow the adversarial DR to perform a series of unauthorized actions: obtaining a valid TokenAddr, downloading the corresponding encrypted token from the Tangle, and decrypting the encrypted token using the HIBE keys. Furthermore, armed with the appropriate keys, the adversary could effortlessly pass the challenge-response phase, typically a robust line of defense against unauthorized access.

**Figure 29:**
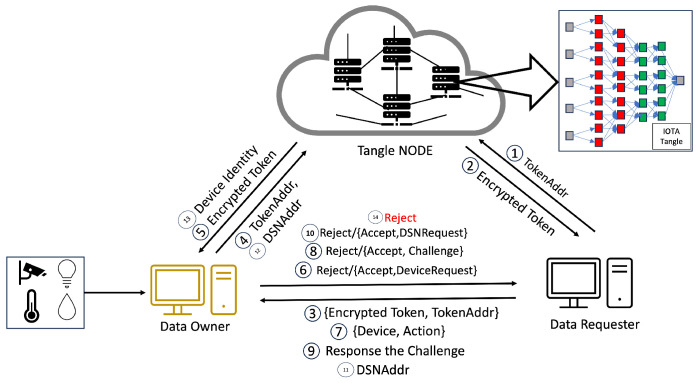
Security response to compromise of authority.

In our system, the last-word challenge (LWC), as illustrated in step 10 in Figure 29, played a critical role in detecting whether a compromised authority registered a DR. This mechanism involved the verification of the DSN within the Tangle. Each DA intending to upload a DR’s DSN had to obtain signatures from other DAs within their domain. In cases where a DA was compromised, it failed to respond correctly to the LWC, thereby preventing other DAs from endorsing it with their signatures.

A potential workaround for a compromised DA was to forge signatures within the DSN. However, this approach was effectively countered when another DA downloaded the DSN from the Tangle. Upon decrypting the DSN, the DA verified the signatures from other domain authorities. The inevitable outcome in such cases was the invalidation of the signatures, leading to the rejection of the request in Step 14 in Figure 29. This process ensured a robust layer of security, effectively mitigating the risks associated with a high-level authority compromise.

## 7. Conclusions

### 7.1. Summary

The comprehensive performance evaluation of our system, as delineated in Section 6, offers significant insights into its operational efficiency and security robustness. Our hierarchical system evaluation highlights the scalability challenges and the need for platform-specific optimizations in IoT environments, with significant execution time differences observed on AWS and the Raspberry Pi 4, particularly in access rights delegation, access rights authorization, and access rights verification processes. Despite the IHIBE scheme taking slightly longer time than the ZNSK scheme, only IHIBE among them is suitable for hierarchical organization. Our security assessment confirms the robustness of the challenge-response method in safeguarding system integrity against various adversarial scenarios, including encrypted token theft and authority compromise.

### 7.2. Future Work

Furthermore, it is advised to thoroughly investigate the system’s resistance to potential security risks and attacks. A detailed security evaluation is essential for establishing a strong defense against hostile threats. Identifying vulnerabilities and developing targeted strategies fortifies the system’s hierarchical structure.

The incorporation of newer technology or extra cryptography approaches is something that should be taken into account in the development of a more comprehensive security framework. Examining the integration of techniques like homomorphic encryption and zero-knowledge proofs could improve the system’s privacy-preserving features.

## Figures and Tables

**Table 1:** Optimal depths of mechanisms relative to service requirements in IoT systems.

Service	Traffic Rate	Tolerable Delay	Depth on AWS	Depth on Raspberry Pi 4
Structural health	1 pct/10 min/device	30 min	Up to 640	Up to 640
Waste management	1 pct/1 h/device	30 min	Up to 640	Up to 640
Air quality monitoring	1 pct/30 min/device	5 min	Up to 320	Up to 160
Noise monitoring	1 pct/10 min/device	5 min	Up to 320	Up to 160
Traffic congestion	1 pct/10 min/device	5 min	Up to 320	Up to 160
City energy consumption	1 pct/10 min/device	5 min	Up to 320	Up to 160
Automation and salubrity of public buildings	1 pct/10 min/device	5 min	Up to 320	Up to 160

## Data Availability

Data are contained within the article.

## Abbreviations

The following abbreviations are used in this manuscript:

CP-ABE	Ciphertext policy attribute-based encryption
IBE	Identity-based encryption
HIBE	Hierarchical identity-based encryption
IHIBE	IOTA with Hierarchical identity-based encryption
DO	Data owner
DR	Data requesters
DA	Domain authority
CA	Central authority
PP	Public parameter
MSK	Master secret key
SK	Secret key
DR	Data requester
